# The role of Rho/ROCK in epileptic seizure-related neuronal damage

**DOI:** 10.1007/s11011-022-00909-6

**Published:** 2022-02-04

**Authors:** Zhihan Wang, Dabin Ren, Ping Zheng

**Affiliations:** 1 Department of Neurosurgery, Shanghai Pudong Hospital, Fudan University Pudong Medical Center, Shanghai, 201399 China; 2 Department of Neurusurgery & Key Laboratory, Shanghai Pudong New area People’s Hospital, Shanghai, 201299 China

**Keywords:** Rho/ROCK, Epileptic seizure, Neuronal damage

## Abstract

Epilepsy is one of the most severe neurological disorders characterized by spontaneous recurrent seizures. Although more than two-thirds of patients can be cured with anti-epileptic drugs (AEDs), the rest one-third of epilepsy patients are resistant to AEDs. A series of studies have demonstrated Rho/Rho-associated kinase (ROCK) pathway might be involved in the pathogenesis of epilepsy in the recent twenty years. Several related pathway inhibitors of Rho/ROCK have been used in the treatment of epilepsy. We searched PubMed from Jan 1, 2000 to Dec 31, 2020, using the terms "epilepsy AND Rho AND ROCK" and "seizure AND Rho AND ROCK". We selected articles that characterized Rho/ROCK in animal models of epilepsy and patients. We then chose the most relevant research studies including *in-vitro*, *in-vivo* and clinical trials. The expression of Rho/ROCK could be a potential non-invasive biomarker to apply in treatment for patients with epilepsy. RhoA and ROCK show significant upregulation in the acute and chronic stage of epilepsy. ROCK inhibitors can reduce the epilepsy, epileptic seizure-related neuronal death and comorbidities. These findings demonstrate the novel development for diagnosis and treatment for patients with epilepsy. Rho/ROCK signaling pathway inhibitors may show more promising effects in epilepsy and related neurological diseases.

## Introduction

Rho/Rho-associated kinase (ROCK) signaling pathway induces cytoskeleton recombination, cell migration, stress fiber formation and vascular and tissue permeability (Deng et al. 2019). The contraction of microtubules is related to various physiological functions such as growth. Diabetes, kidney disease, eye disease, tumor, heart disease, hypertension, radiation injury and leukemia are related to Rho/ROCK signaling pathway activation(Deng et al. 2019). In addition, it is involved in spinal cord injury(Kishima et al. 2021) and traumatic brain injury and Alzheimer's disease and other neurological diseases(Raad et al. 2012). Recently, studies have shown that Rho/ROCK signal pathway suppresses epilepsy, the collapse of growth cone and the growth of axon (Çarçak et al. 2018). Rho/ROCK, as a drug development target for neurotoxic diseases and excitotoxic, more and more researchers pay attention to this signal pathway. However, there is currently no review article regarding the Rho/ROCK pathway in seizure or epilepsy.

Rho is a small guanosine triphosphate (GTP) enzyme and belongs to Ras superfamily. Rho GTPase is divided into several subfamilies. The most widely studied member is Ras homologous gene family A (Ras homolog gene family, member A, RhoA), Similar to Ras, Rho consists of molecular switches controlled by regulatory proteins, including guanosine nucleotides exchange factor (guaranine nucleotide exchange factor, GEF), GTPase activating protein (Gap) and GDP dissociation inhibitor, GDI). GDI exists in the cytosol in the form of GDP GDI, making Rho GTP enzymes are isolated to convert Rho into an inactive form(Keller et al. 2019). Among them, RhoA mediated formation of stress fibers and adhesive spots(Kalpachidou et al. 2019), Rac1 induced lamellar pseudopodia and formation of membrane fold, formation of filiform pseudopodia induced by Cdc42(Bai et al. 2019). In addition, Rho GTP regulates actin cytoskeleton in dendritic spines. It plays an important role in morphogenesis and synaptic plasticity.

ROCK, also known as Rho kinase, belongs to serine / threonine protein kinase, whose molecular weight is about 160 kDa(Hensel et al. 2017). Research shows that ROCK has two isotypes: ROCK I and ROCK II(Gu et al. 2020). ROCK I is mainly in the heart, lung and other non-neuronal tissues. ROCK II is mainly expressed in brain, spinal cord and muscle, and increases with age(Yamaguchi et al. 2017). Rho-ROCK pathway has been previously found to be an inhibitory signal to prevent the axonal growth in CNS injury, and Rho-ROCK inhibitors can facilitate axonal regeneration(Kubo and Yamashita 2007). The Rho-ROCK pathway has been widely investigated in spinal cord injuries(Luo et al. 2021), stroke(Lee et al. 2014) and Alzheimer’s disease (AD)(Hamano et al. 2020), and traumatic brain injury (TBI)(Mulherkar and Tolias 2020). However, its role in epileptic seizure, especially on the seizure-related neuronal death has not been studied comprehensively. Therefore, this study aims to explore the role of Rho/ROCK in epileptic seizure-related neuronal damage literally.

## Methods

We searched PubMed from Jan 1, 2000 to July 31, 2021, using the terms "epilepsy AND Rho AND ROCK" showing 9 results and "seizure AND Rho AND ROCK" showing 11 results. And there are no review articles regarding the Rho/ROCK in seizure or epilepsy. Among them, four articles are replicated and finally we identified 16 articles that characterized Rho/ROCK in animal models of epilepsy and patients. We then chose the most relevant research papers that could be relevant to epilepsy including *in-vitro* or *in-vivo* studies and clinical trials.

## Results

### Epilepsy-relevant Rho / ROCK alterations

RhoA belongs to the Ras superfamily of G-proteins and is actively expressed in different cells, including neurons and astrocytes(Katayama et al. 2011). Several studies have shown Rho is involved in neurite outgrowth and synaptic plasticity(Fujita and Yamashita 2014). RhoA is activated in the hippocampus after brain insults and kainic acid (KA)-induced epilepsy(Dubreuil et al. 2006). In the injured CNS, ROCK activation increase neurite retraction(Huang et al. 2017), while ROCK inhibition improve cellular and axonal regeneration(Huang et al. 2017). The ROCK inhibitors, fasudil and Y-27632, were accordingly identified as an intracellular calcium antagonist and applied to treat cerebral vasospasm following subarachnoid hemorrhage(Jeon et al. 2013).

Given that RhoA/ROCK plays a critical role in the pathophysiology of CNS diseases, the development of therapeutic agents targeting this pathway contributes to the treatment of CNS diseases. The RhoA/ROCK pathway mediates the effects of myelin-associated axon growth inhibitors—Nogo, myelin-associated glycoprotein (MAG), oligodendrocyte-myelin glycoprotein (OMgp), and repulsive guidance molecule (RGM). Blocking RhoA/ROCK signaling can reverse the inhibitory effects of these molecules on axon outgrowth, and promotes axonal sprouting and functional recovery in animal models of CNS injury(Fujita and Yamashita 2014). As myelin and axon are the main white matter in the brain, the abnormal expression of RhoA/ROCK results in the impaired structural alterations, and further cause the functional deficits in epilepsy and other neurological disorders. We previously showed that, epileptic rodents (a epilepsy model introduced by Kainic acids) display disturbed corpus callosum shown by reduced fractional anisotropy (FA) value with diffusion tensor imaging (DTI), however, we did not check the expression of RhoA or ROCK expression or activity in these rodents(Liu et al. 2016). A recent study by Xiang et al. found inhibition of RhoA/ROCK signaling pathway by fasudil protects against kainic acid-induced neurite injury(Xiang et al. 2021).

### Opportunities for Rho/ROCK inhibitors in epilepsy treatment

#### Rho/ROCK inhibitors in epilepsy

Researchers have found Rho/ROCK pathways with the potential for becoming targets in order to understand more about epilepsy. Basic treatment to examine the effectiveness of suppressing Rho/ROCK in reverse epilepsy should be validated through a clinical translation test in epileptic patients. Y-27632, a ROCK inhibitor, reduces hippocampal RhoA and ROCK2 expression in KA-induced epilepsy mice, while Y- 27,632 also facilitated neurite formation in excitotoxicity by glutamate in vitro. These findings suggest that ROCK inhibitor mediates neurite growth and protects neurons from epileptic seizure-induced cell death(Jeon et al. 2013). However, another research found Y-27632 was not suitable for post status-epilepticus (post-SE), as it had a detrimental effect on these rodents. Nazim et al. found that Y-27632 also has a worsening effect when used in post-SE rats at the chronic stage via reducing reactive astrogliosis and inducing neuronal death(Kourdougli et al. 2015). This might be due to different acquired epilepsy models induced by different chemicals like kainic acid versus pilocarpine. As such, in Genetic Absence Epilepsy Rats from Strasbourg (GAERS), a traditional absence model, Y-27632 and Fasudil could reduce Spike- And-Wave Discharges (SWDs) in these rats. Compared with control rats, GAERS rats developed higher RhoA activity in the somatosensory cortex but not in the thalamus or hippocampus (limbic system). Systemic administration (intraperitoneal) of Y-27632 and fasudil partly reduced both frequency and duration of an absence seizure attack. However, local brain administration caused a widespread suppressive effect on the total seizure frequency and duration as well. Therefore, Rho/ROCK signaling might be implicated in the mechanism of absence seizure(Çarçak et al. 2018). Besides the direct pharmaceutical target, the Rho/ROCK pathway could also be regulated by non-pharmaceutical methods. Low-Frequency electrical stimulation has been shown to effectively treat the rat epilepsy model via the RhoA/ROCK signaling pathway. In comparison with the epilepsy group, the seizure frequency, duration, and seizure power, the mRNA and protein expressions of IL-1β and IL-1R1, the expressions of RhoA and ROCK I proteins, and the ratio of RhoA protein between membrane and cytosol decreased in the electrical stimulation group(Liu et al. 2018).

#### Rho/ROCK inhibitors in epilepsy-related comorbidity

Patients with epilepsy would also develop comorbidities like cognitive dysfunction and depression(Shultz et al. 2015; Liu et al. 2016). Y-27632 significantly reduced the immobility time of rats and increased swimming and climbing duration in the forced swimming test(Inan et al. 2015). Therefore, Y-27632 demonstrates antidepressant-like activity in rats. In addition, ROCK inhibitor Fasudil is reported to improve cognition in SE rats tested by Morris Water Maze(Song et al. 2019). Their findings suggest that Fasudil given at the onset could improve cognitive function by decreasing neuronal damage, and also reducing EEG discharges, indicating the roles for the Rho/ROCK signaling pathway in the mechanisms of brain damages in SE. The RhoA/ROCK signaling pathway is a potential target for the treatment of epilepsy-induced brain damages.

We have previously shown that tau phosphorylation is involved in epileptogenesis. Hyperphosphorylated tau (p-tau) can decrease the seizure threshold in several animal models of epilepsy (kindling amygdala model, post-status epilepsy and post-traumatic epilepsy)(Liu et al. 2016). Based on that, we target the p-tau with pharmacological and genetic manipulation to prevent the neurodegeneration and reduce spontaneous seizure in these preclinical models(Shultz et al. 2015). Recently, it is found that ROCK inhibitors decrease the p-tau in an AD model for tauopathies, and inhibition of Rho/ROCK pathway is able to activate autophagy and proteasome to degrade tau proteins through inactivating glycogen synthase kinase 3β, cyclin-dependent kinase 5, and caspase and further activating protein phosphatase 2A(Hamano et al. 2020).

The imbalance between Hyperexcitability and GABAergic neurons alterations is not only considered to be a major pathophysiological basis in epilepsy. It also occurs in the disease named X-linked intellectual disability. In these cases, ROCK inhibition could rescue the hippocampal hyperexcitability as well(Busti et al. 2020), which indicates Rho/ROCK inhibitor is able to treat epilepsy-related neuronal damage.

### The challenge of microRNA in epilepsy

MicroRNAs (miRNAs) are a family of non-coding RNA with a length of 18–22 nucleotides, and are widely expressed in several organisms(Zheng et al. 2019). They play a role as post-transcriptional regulators by binding to several mRNAs causing translational repression or target degradation and genetic silencing (Ubhi et al. 2014). Currently, abnormal alterations of miRNAs have been identified in epilepsy, such as miR-218 and miR-204(Kaalund et al. 2014). Serum profiles of the miRNA of 30 epileptic patients and 30 healthy control identified 4 miRNAs were significantly upregulated (let-7d-5p, miR-106b-5p, miR-130a-3p, and miR-146a-5p), whereas 6 miRNAs (miR-15a-5p, -144-5p, -181c-5p, -194-5p, -889-3p, and novel-mir-96) were downregulated. The authors further validated miR-144 and miR-96 were both downregulated with RT-PCR(Wang et al. 2016) (Table 1).

**Table 1:** Summary of targeting ROCK or miRNA in models of epilepsy

Reference No	Treatment method	Treatment time	Animal Model	Pathology & Physiology
20	ROCK inhibitor: Y-27632	1 day before modeling	Glutamate in HT22 cells KA in mice (post-SE)	Decreased death in hippocampal neurons
21	ROCK inhibitor: Y-27632	6 weeks after modeling	Rat pilocarpine model (post-SE)	Increased neuronal death in CA3 Increased Mossy Fiber Sprouting
4	Y-27632 and Fasudil	NA	GAERS	Decreased seizure frequency and duration
22	Low frequency electrical stimulation	1 day after modeling	Hippocampal electrical stimulation	Decreased seizure frequency and duration
26	ROCK inhibitor: Fasudil	1 or 5 days after modeling	Pilocarpine Rats (post-SE)	Decreased neuronal damage Increased cognitive function Decreased EEG discharge
33	NA	NA	Pilocarpine rats (post-SE) Epilepsy patients	Increased expression of Rac1
42	miR-134 oligonucleotide	NA	KA in rats (post-SE)	Decreased neuronal death in CA3 Decreased Mossy Fiber Sprouting

As miRNAs are able to inhibit the transcription of mRNAs like Rho or ROCK, we did the verification based on the relationship between decreased miRNAs in epilepsy and Rho or ROCK. We found that RHOA is a target for has-miR-144 from miRDIP website(Tokar et al. 2017) and this is consistent with the findings that the expression level of miR-144 is reduced and RHOA/ROCK is upregulated in epilepsy. Furthermore, we applied the Targetscan analysis(Shin et al. 2010) to link miRNAs and RHOA/ROCK pathways and found the target gene of miR-96 is RAC1 (Table 2), while the target gene of miR-144 is ROCK1 (Table 3) and ROCK2 (Table 4). However, the exact relationship between them needs to be verified in luciferase experiments.

**Table 2:**
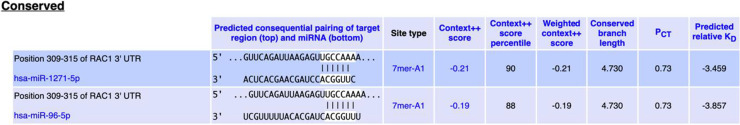
The conserved binding for miR-96-5p and RAC1

**Table 3:**
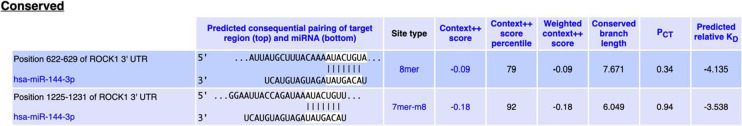
The conserved binding for miR-144 and ROCK1

**Table 4:**
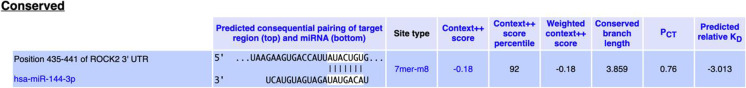
The conserved binding for miR-144 and ROCK2

**Fig. 1:**
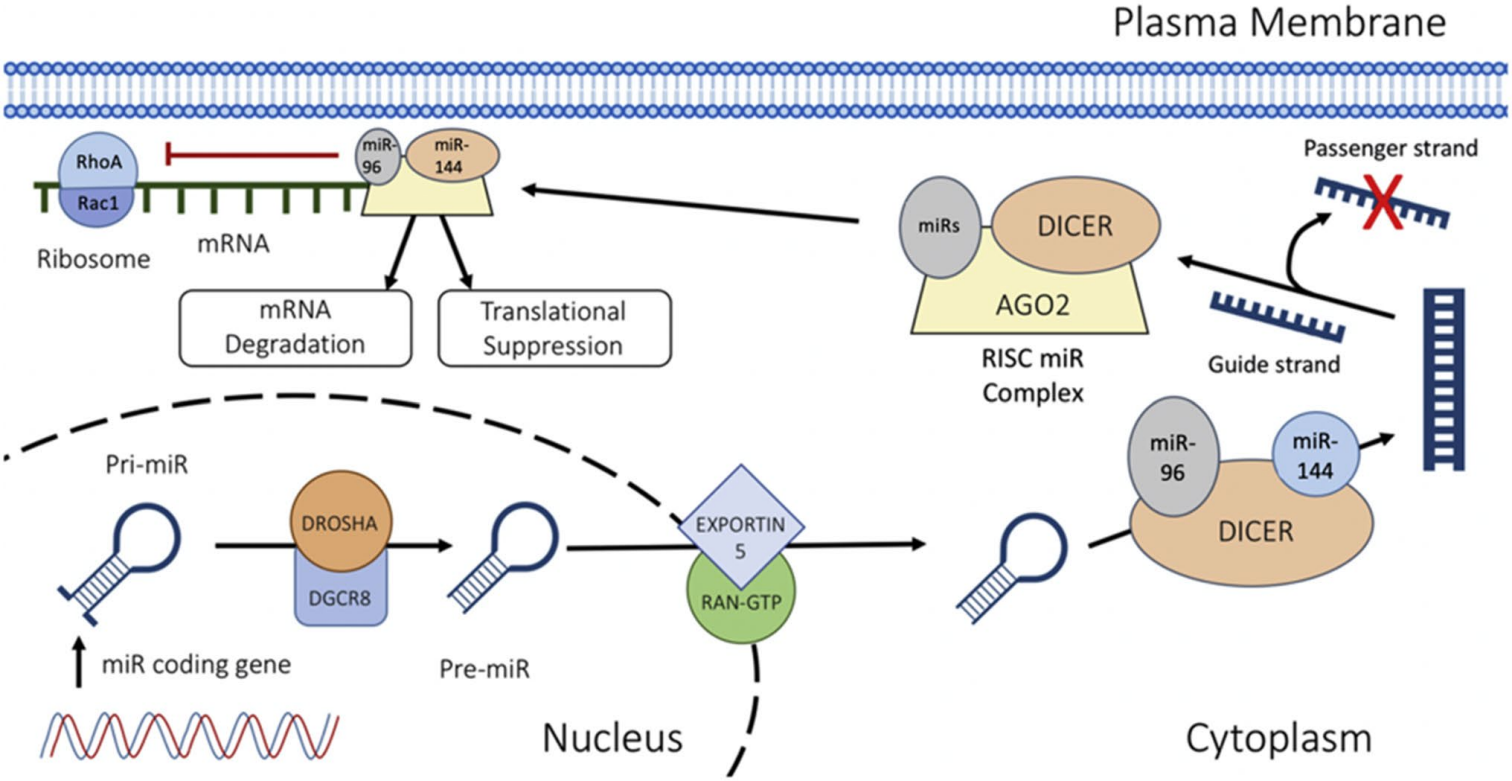
Biogenesis of miR-96 and miR-144. The miRNA is transcribed by RNA polymerase II to form a primary miRNA. The RNase III enzyme DROSHA cuts the single-stranded RNA/double-stranded RNA (ssRNA/dsRNA) junction to create a preliminary-miRNA (Pre-miR-96 or Pre-miR-144). Pre-miRNA transports from nucleus into cytoplasm via Ran-GTP and Exportin 5 family proteins. Then pre-miRs can be modified by DICER. The guide strand is then incorporated into the RNA-induced silencing complex (RISC) together with AGO2, where it leads the complex toward target mRNA transcripts to prevent the transcription of these mRNAs

A current study demonstrated that downregulated RAC1 decreased recurrent seizures in animal models of epilepsy, and found that RAC1-GTP was exclusively implicated in the mechanism of epilepsy(Li et al. 2016). However, these authors did not further explore the upstream mechanism in reduced RAC1 activity in epilepsy and the downstream pathway of RAC1 in epilepsy also remains to be studied. In addition, miR-96 plays an important role in Rac1 signaling pathway in a mouse model of inherited retinal degeneration(Palfi et al. 2016). The relationship between miR-96 and Rac 1 was verified both in-vitro and in vivo with a luciferase assay. They also reported miR-96 is involved in cell adhesion, signal transduction and neuronal apoptosis (Palfi et al. 2016). In recent years, there are tons of researches on miR-96 mediating several diseases, such as hepatocellular carcinoma, urothelial carcinoma and breast cancer(Yamada et al. 2011; Li and Wang 2018). However, there are few kinds of research on the role of miR-96 in epilepsy. These findings indicate that both miR-144 and miR-96 might be a potential target for the treatment of epilepsy, and become worthwhile to investigate in the future study (See Fig. 1). As the Rho/ROCK pathway was abnormally activated in epilepsy, it would be proposed that the miR-96 and miR-144 expression were downregulated in epilepsy. And mimics of miR-96 and miR-144 might be helpful in inhibiting epileptic seizure and reducing the neuronal loss.

### Other biomarkers in epilepsy

Two of the key goals of therapy development in the epilepsy field are to develop new treatments that are effective in patients with drug-resistant seizures or are antiepileptogenic/disease modifying(Galanopoulou et al. 2012). At least 30% of patients with epilepsy are unable to achieve seizure control with currently available anti-epilepsy drugs (AEDs), and the introduction of multiple new drugs over the last two decades has done little to change this(Mula 2020). All current AEDs only suppress seizures, with none confirmed to stop or reverse the process that converts a healthy brain into an epileptic brain, that is, epileptogenesis(Löscher and Brandt 2010).

There is emerging evidence that targeting miRNA-based mechanisms can have both antiseizure and antiepileptogenic efficacy, and has the potential to represent a novel approach for new therapy development for epilepsy patients. Recently, several articles regarding the biomarkers for epilepsy have been published. Sueri et al. gave a brief overview on imaging markers, electrophysiological biomarkers and serological biomarkers for epilepsy, and they identified that promising serological biomarkers of epileptogenicity include inflammation molecules and miRNAs(Sueri et al. 2018). This is consistent with our findings that the role of miR-96 and RhoA pathways in epilepsy-associated inflammation. Henshall’s group also identifies that blood miRNA as molecular biomarkers of epilepsy with encouraging receiver-operating characteristic (ROC) curve(Enright et al. 2018). In addition, they reviewed the miR-134 targeted therapy in epilepsy, which has a disease-modifying effect. MiR-134 has been found in both patients and animal models of epilepsy. BDNF is a target of miR-134, which is decreased in epilepsy and they further found that miR-134 oligonucleotide could increase the expression of BDNF and reduce the frequency and duration of seizure in animal models(Morris et al. 2019). Dr. Bauer recently reviewed the role of miRNAs in epilepsy and addressed that future research should focus on the feasibility of miRNAs as biomarkers for epilepsy in humans as well as treatment response(Bauer et al. 2020). Meanwhile, few studies could independently validate miRNAs as biomarkers, with highly discrepant results. This indicates that there is still a long way to form a miRNA-targeted therapy in epilepsy.

## Conclusion

In conclusion, our study revealed that RhoA and ROCK signaling abnormally activated in epilepsy and cause epileptic seizure-related neuronal death. Targeting RhoA and ROCK has been shown to have a neuroprotection both in-vitro and in-vivo (Table 1). Further investigation of the cell-specific mechanism and miRNA-based therapy should be more scrupulously and profoundly performed with a larger cohort to provide a promising clinical application in treatment for patients with epilepsy.

## Data Availability

The datasets supporting the conclusions of this article are available from the corresponding author.
